# Benchmarking the next generation of homology inference tools

**DOI:** 10.1093/bioinformatics/btw305

**Published:** 2016-06-01

**Authors:** Ganapathi Varma Saripella, Erik L. L. Sonnhammer, Kristoffer Forslund

**Affiliations:** ^1^Science for Life Laboratory, Stockholm Bioinformatics Center, Department of Biochemistry and Biophysics, Stockholm University, Stockholm SE-10691, Sweden; ^2^European Molecular Biology Laboratory, Structural and Computational Biology Unit, Heidelberg 69117, Germany

## Abstract

**Motivation:** Over the last decades, vast numbers of sequences were deposited in public databases. Bioinformatics tools allow homology and consequently functional inference for these sequences. New profile-based homology search tools have been introduced, allowing reliable detection of remote homologs, but have not been systematically benchmarked. To provide such a comparison, which can guide bioinformatics workflows, we extend and apply our previously developed benchmark approach to evaluate the ‘next generation’ of profile-based approaches, including CS-BLAST, HHSEARCH and PHMMER, in comparison with the non-profile based search tools NCBI-BLAST, USEARCH, UBLAST and FASTA.

**Method:** We generated challenging benchmark datasets based on protein domain architectures within either the PFAM + Clan, SCOP/Superfamily or CATH/Gene3D domain definition schemes. From each dataset, homologous and non-homologous protein pairs were aligned using each tool, and standard performance metrics calculated. We further measured congruence of domain architecture assignments in the three domain databases.

**Results:** CSBLAST and PHMMER had overall highest accuracy. FASTA, UBLAST and USEARCH showed large trade-offs of accuracy for speed optimization.

**Conclusion:** Profile methods are superior at inferring remote homologs but the difference in accuracy between methods is relatively small. PHMMER and CSBLAST stand out with the highest accuracy, yet still at a reasonable computational cost. Additionally, we show that less than 0.1% of Swiss-Prot protein pairs considered homologous by one database are considered non-homologous by another, implying that these classifications represent equivalent underlying biological phenomena, differing mostly in coverage and granularity.

**Availability and Implementation:** Benchmark datasets and all scripts are placed at (http://sonnhammer.org/download/Homology_benchmark).

**Contact:** forslund@embl.de

**Supplementary information**: Supplementary data are available at *Bioinformatics* online.

## 1 Introduction

Modern molecular biology relies on evolutionary conservation of properties between entities such as genes and proteins that are homologous, i.e. share descent from a common ancestor. As a historical property homology is unobservable but can be inferred from statistically significant similarity under the proper conditions (Henikoff and Henikoff, 1992). Through homology relationships (and within them, specifically orthology relationships where common ancestry dates back to a species diversification rather than a gene duplication), insights into molecular function of whole sequences (Bork *et al.*, 1998) or specific sites (Yao *et al.*, 2003), 3D structure (Chothia and Lesk, 1986), (Todd *et al.*, 2001) or context such as regulation can be transferred. Such transfer of results from direct experimentation to the components of the vast number of genomes for which only molecular data is available, courtesy of ‘next-generation’ nucleotide sequencing techniques, means homology inference forms a mainstay in bioinformatics research as well as in its applications in organismal, clinical and evolutionary biology. These methods started with the Smith–Waterman algorithm (Smith and Waterman, 1981) for exact computation of the minimal number of changes needed to convert one sequence into another. Gradually more complex probabilistic models were developed taking implicitly into account the structural constraints and codon properties of nucleic acid substitutions, insertions and deletions. Sequence alignment/homology search/homology scoring methods quickly became overwhelmed by computational complexity as database sizes increased, prompting development of heuristic tools like FASTA (Pearson and Lipman, 1988) or NCBI-BLAST (Altschul *et al.*, 1990) which function fast enough to screen the whole of the known sequence universe for similarity to a novel uncharacterized query.

With heuristic approaches come increased risk of error, and given the potential importance of downstream applications such as function prediction, the need becomes clear to properly evaluate the reliability of homology inference tools. This is in itself not a trivial problem, since such benchmarking ideally should involve a ‘gold standard’ where homology status—whether shared common ancestry holds or not—should be known with perfect certainty, which is in principle never the case.

The existence of well-conserved ‘building blocks’ of protein sequence and structure, as in domain/gene families where in many cases subtle sequence similarity is supported by clearer similarity of the slower-evolving protein 3D structure (Chothia and Lesk, 1986), makes for a potential workaround. Early on a preferred benchmark was evaluating single-domain sequences from same or different structural superfamilies as a proxy for certain positive or negative homology status (Chandonia *et al.*, 2004). This disregards the theoretical and practical difficulties which arise when domain rearrangement or other forms of horizontal evolution causes mosaic gene lineages (Vogel *et al.*, 2004), where different regions have different homologs, which is a complexity that the approach described here also disregards. More tractable difficulties for homology inference arises either when sequences have diverged too far (risk of failing to detect homology) or are unexpectedly similar due to similar sequence composition biases and/or low-complexity region features (risk of falsely inferring homology).

Several issues in creating benchmarking datasets have been discussed earlier (Aniba *et al.*, 2010). Low-complexity regions occur relatively seldom within well-characterized single-domain sequences, but will occur elsewhere in proteins, making single-domain benchmarks underestimate the risk of false positives in genome-scale homology inference applications. To remedy this, we previously (Forslund and Sonnhammer, 2009) described an approach for generating ‘gold standard’ test cases for homology inference by selecting pairs of multi-domain proteins where either all corresponding domains match at the super-family/clan level (positive gold standard) or where none of them do (negative gold standard). Using this approach, we compared different low-complexity filter settings for the NCBI-BLAST homology search tool, and found that compositional adjustment of score matrices allowed minimization of false positives, though sometimes at the price of truncated alignments.

More recent developments in homology inference involve profile-based tools for detecting remote homologies, using profile-specific score matrices (PSSMs) (Gribskov *et al.*, 1987), Hidden Markov Models (HMMs) (Eddy, 1998) or other techniques (Altschul *et al.*, 1997, Altschul and Koonin, 1998). These ‘next-generation’ homology search tools may offer greater sensitivity and search speed (Elofsson, 2002), and because of these promises, the need for formal evaluation of their reliability arises (Müller *et al.*, 1999). Consequently, we expanded on our previous benchmark approach to construct an updated evaluation dataset, then tested the latest versions of the ‘next-generation’ homology search tools for precision, accuracy and speed.

Additionally, we applied our benchmarking method to all three major domain family databases: SUPERFAMILY (extending SCOP, Fox *et al.*, 2014; Gough *et al.*, 2001; Hubbard *et al.*, 1999; Oates *et al.*, 2015), Gene3D (extending CATH, Lees *et al.*, 2013) and Pfam (Finn *et al.*, 2014), where previously only Pfam was used. This was done with the intent that the similarity of benchmark results derived from different databases would provide a test of to what extent, beyond differences in scope or coverage, that these resources, built from different types of data and using different curation protocols, reflect the same underlying evolutionary entities seen through different definition schemes, a question which has been raised in some recent studies (Csaba *et al.*, 2009).

## 2 Methods

**Fig. 1.**
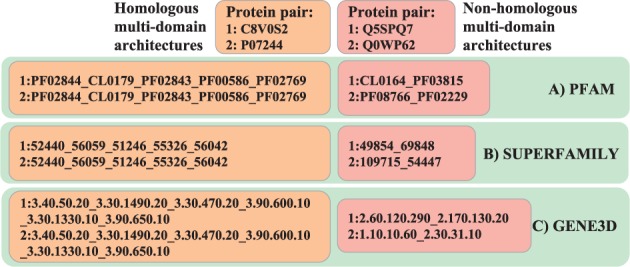
Diagram illustrating how multi-domain homologous and non-homologous protein pairs were selected from the three databases Pfam (with clans), SUPERFAMILY and Gene3d. Pfam architectures were considered at the Clan level by replacing Pfam domain IDs with Clan IDs where defined. Architectures are listed as consecutive domain identifiers separated by an underscore (_). Only architectures with two or more domains were considered

As previously described (Forslund and Sonnhammer, 2009), pairs of multi-domain proteins are seen as homologous for the purpose of the benchmark if their domains, in consecutive order, belong to the same family or clan (in the case of Pfam) or the same superfamily (in the case of Gene3D or SUPERFAMILY). If no domain in the first protein is part of the same family/clan/superfamily as any domain in the second protein, the pair is instead considered non-homologous for the purpose of the benchmark. Protein pairs where neither condition held are considered potentially ambiguous and not used. All domain architectures and sequences were acquired from the source databases (version 28.0 of Pfam, version 1.75 of SCOP/SUPERFAMILY, version 3.5.0 of Gene3D), retrieving all domain matches via v53.0 of the InterPro database (Mitchell *et al.*, 2015), restricting the analysis to sequences present in SwissProt (UniProtKB/Swiss-Prot, downloaded on August 24 2015). To account for incompleteness of present domain annotations, any sequence was discarded for which at least fifty consecutive residues were not assigned to a protein domain, as has been done in previous studies (Forslund *et al.*, 2008; Gough, 2005). Figure 1 displays examples of homologous and non-homologous pairs based on domain architectures from each source database.

For the specific benchmark dataset, all sequences from a specific set of genomes were included, chosen to represent the span of (model organism) diversity while remaining small enough to be manageable (Sayers *et al.*, 2012)—see Supplementary Table S1 for details on this set of genomes. Within this set of sequences, for each domain database, we considered each distinct protein multi-domain architecture (PMDA) separately. In the case of Pfam, consecutive repeat/motif-type domains, were collapsed to a single instance as in Forslund and Sonnhammer (2009), because repeat numbers are highly variable. Protein pairs were sampled to avoid biasing the analysis towards highly populated gene families. For each architecture, one (if only one exists) or two proteins with that architecture were randomly chosen from each genome in the benchmark, and the set of pairs these proteins define were included, aiming to ensure both within-species and across-species homologies at different evolutionary distance was sampled for each architecture. Negative test cases (pairs of non-homologous proteins) were sampled by choosing a protein from the architecture in question and another randomly selected architecture meeting the criterion for non-homology, i.e. no domains shared in any order even at clan or superfamily level, until there were as many negatives as positives for each PMDA. See Supplementary Table S2 for details on the number of pairs generated for the final benchmark dataset.

**Fig. 2.**
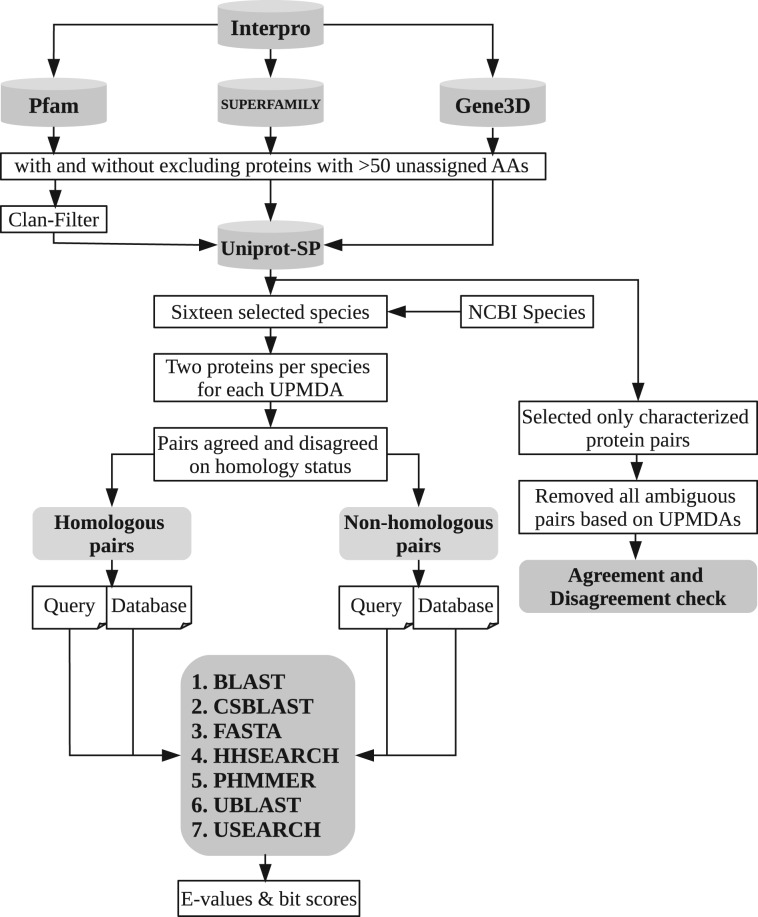
Flowchart illustrating the construction of the benchmark dataset. Protein pairs were selected from the UniProt-SP database based on three domain databases, removing any proteins with more than 50 consecutive residues not assigned to any domains as an initial filtering step. The dataset was restricted to 16 selected species. Pairs of proteins were subsequently retained if definable as clearly homologous or clearly non-homologous based on our domain architecture criterion, for all three of the compared domain databases

For each protein pair evaluated, each pair was aligned (i.e. one protein used as database, one as query) using each of the profile-based homology search tools CS-BLAST (Biegert and Söding, 2009), HHSEARCH (Söding, 2005) and PHMMER (Finn *et al.*, 2011) as well as the non-profile based NCBI-BLAST (Boratyn *et al.*, 2013), USEARCH/UBLAST (Edgar, 2010) and FASTA (Pearson and Lipman, 1988) for comparison. All methods were run with default parameters where not otherwise noted (see Supplementary Table S3 for details). The recently developed DELTA-BLAST (Boratyn *et al.*, 2012) was omitted, because it relies on a database of sequence families aside from what is provided at runtime via query and search database input. Similarly tools relying on iterative searches to build intermediate profiles from additional database sequences (e.g. PSI-BLAST; Altschul *et al.*, 1997; or CSI-BLAST) were not included, since their performance depends strongly on the number of iterations and the composition of the database relative to the query, making their evaluation in the present pairwise context difficult. While HHsearch primarily is intended for use with multiple-sequence queries, here only its performance with single-sequence queries is evaluated, in line with the other methods tested—performance thus might be relatively better in a context other than pairwise sequence comparisons. The score of the best high-scoring segment pair (HSP) reported was used, with no attempt to merge together multiple hit fragments, which also matches the common use cases for these tools. Each tool was applied using default settings except for setting any inclusion/reporting thresholds maximally inclusive so as to be able to compare scores also for non-homologous pairs. Even so, some very divergent or non-homologous sequence pairs were not reported even as very poorly-scoring alignments. For these pairs, a maximally poor ‘proxy’ score (bit score = 0) was assigned. When ordering pairs by score for comparisons (e.g. Receiver Operating Curves (ROC)) (Gribskov and Robinson, 1996), in cases of multiple pairs sharing the same score (either the not-found proxy or otherwise), positive and negative cases were evenly distributed within these stretches of pairs so as not to introduce artifacts. See Figure 2 for a schematic of the workflow as a whole.

## 3 Results

### 3.1 Accuracy of different next-generation homology search tools

Three challenging homology benchmarks were set up using protein domain architectures based on either the Pfam, SUPERFAMILY, or Gene3D domain definitions. True homologs were defined as multi-domain protein pairs with identical domain architecture, while true non-homologs were randomly picked as multi-domain proteins pairs with no domain in common. The main advantage of using protein domain databases instead of protein structure databases is that also domains with unknown structure are included, such as domains with low sequence complexity. The benchmarks contain 455, 330 and 339 architectures for Pfam, SUPERFAMILY, or Gene3D, respectively. Protein pairs for these architectures were then sampled from 16 species to build a benchmark set of 5245, 5047 and 5656 homologous protein pairs, respectively, with equal numbers of non-homologous protein pairs sampled as well.

**Fig. 3.**
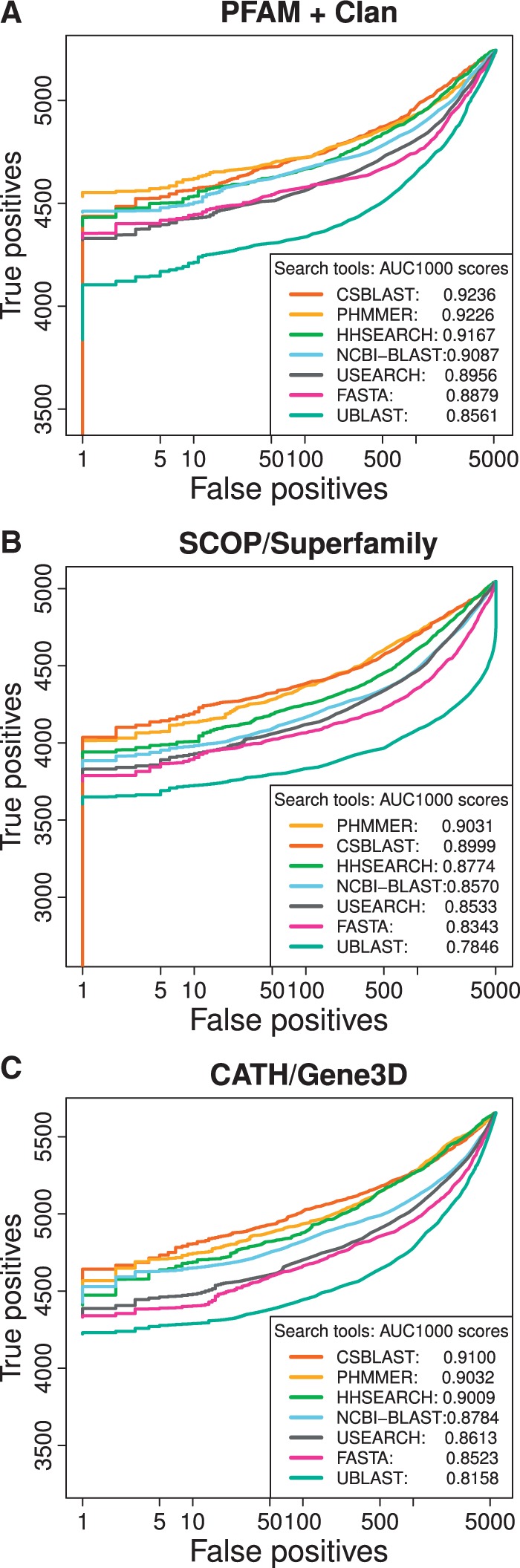
ROC plots showing cumulative true and false positive counts as tested protein pairs (single-sequence query and search database for each pair) are sorted based on the bit scores provided by each method. The curves are ranked by corresponding Area Under Curve scores computed for the first 1000 false positives (AUC1000). Results are shown based on Pfam (**A**), SUPERFAMILY (**B**) and Gene3D (**C**). These benchmarks exclude any proteins with >50AA regions without domain assignments. Supplementary Figure S1A–C show corresponding plots for a dataset where this constraint is removed, leading to the inclusion of many more proteins with disordered regions; the here observed trends were largely replicated (Color version of this figure is available at *Bioinformatics* online.)

In total, seven homology search methods were benchmarked: the three profile search tools CS-BLAST, HHSEARCH and PHMMER, as well as the four single sequence search tools FASTA, NCBI-BLAST, UBLAST and USEARCH. To make a fair comparison, we ran all tools with single sequence queries, that is searching the proteins of each benchmark pair against each other in a 1 to 1 setup. Comparing the accuracy (recall/true positives recovered versus precision/false positives avoided) of the tested search tools on all three benchmarks (Fig. 3A–C) shows that CSBLAST and PHMMER perform best, though all profile-based methods perform similarly. They range in AUC1000 (Area Under Curve for the first 1000 false positives) between 0.89 and 0.92. The classic FASTA method performs considerably poorer at AUC ∼0.83-0.89, with USEARCH only slightly better and UBLAST consistently scoring poorest, which makes sense as these two methods were optimized primarily for speed, but surprisingly the faster tool, USEARCH, is clearly more accurate than the slower UBLAST. Overall, the results were very similar using either Pfam, SUPERFAMILY or Gene3D domains to generate the benchmark data. To investigate whether results are stable also with proteins that contain significant disordered regions, the analysis was also run on a version of the benchmark dataset where pairs of proteins with unassigned regions longer than 50 residues were not excluded, with results shown in Supplementary Figure S1A–C. The same overall trends were replicated. Supplementary Figure S2 show corresponding method performance on the benchmark at different specified *E*-value cutoffs.

### 3.2 Different domain definitions largely agree

How different are the three benchmarks? As they are all mapped to UniProt identifiers, we can compare how often a pair in two benchmarks have the same homology or non-homology status. Restricting to protein pairs present in all three databases controls for difference in coverage, as well as somewhat for differences in hierarchical granularity. Agreement between the databases with respect to homology status reflects the extent to which their differing source data, methodologies and curation efforts uncover the same underlying biological entities, even though it does not guarantee that the domain architectures are identical. As seen in Table 1, the three databases are almost never in opposition on the homology status of shared protein pairs. Inspection of randomly sampled cases of disagreement between the databases under this test indicate they largely correspond to differences in granularity, where the databases differ in how their hierarchies are structured, but where comparison at a higher level would resolve the disagreement.

**Table 1.** Table graphic showing for the three benchmark datasets derived from each database to what extent homologous pairs are homologous, ambiguous or non-homologous in the other three databases

	Database	*Pfam*	*Superfamily*	*Gene3D*
	#Homologous pairs in total:	**147** **570**	**142** **666**	**141** **262**
Also homologous in:	*Pfam*	–	138 084 (96.78%)	136 578 (96.68%)
	*Superfamily*	147 259 (99.78%)	–	141 226 (99.97%)
	*Gene3D*	147 155 (99.71%)	142 478 (99.86%)	–
Ambiguous in:	*Pfam*	–	4582 (3.21%)	4652 (3.29%)
	*Superfamily*	180 (0.12%)	–	4 (0.002%)
	*Gene3D*	284 (0.19%)	188 (0.13%)	–
Non-homologous in:	*Pfam*	–	0 (0%)	32 (0.02%)
	*Superfamily*	131 (0.08%)	–	32 (0.02%)
	*Gene3D*	131 (0.08%)	0 (0%)	–

The three databases generally agree on homology/non-homology of protein pairs under our domain-based definition. Note that ambiguous pairs are not used in the ROC analysis.

### 3.3 Run time evaluation

**Fig. 4.**
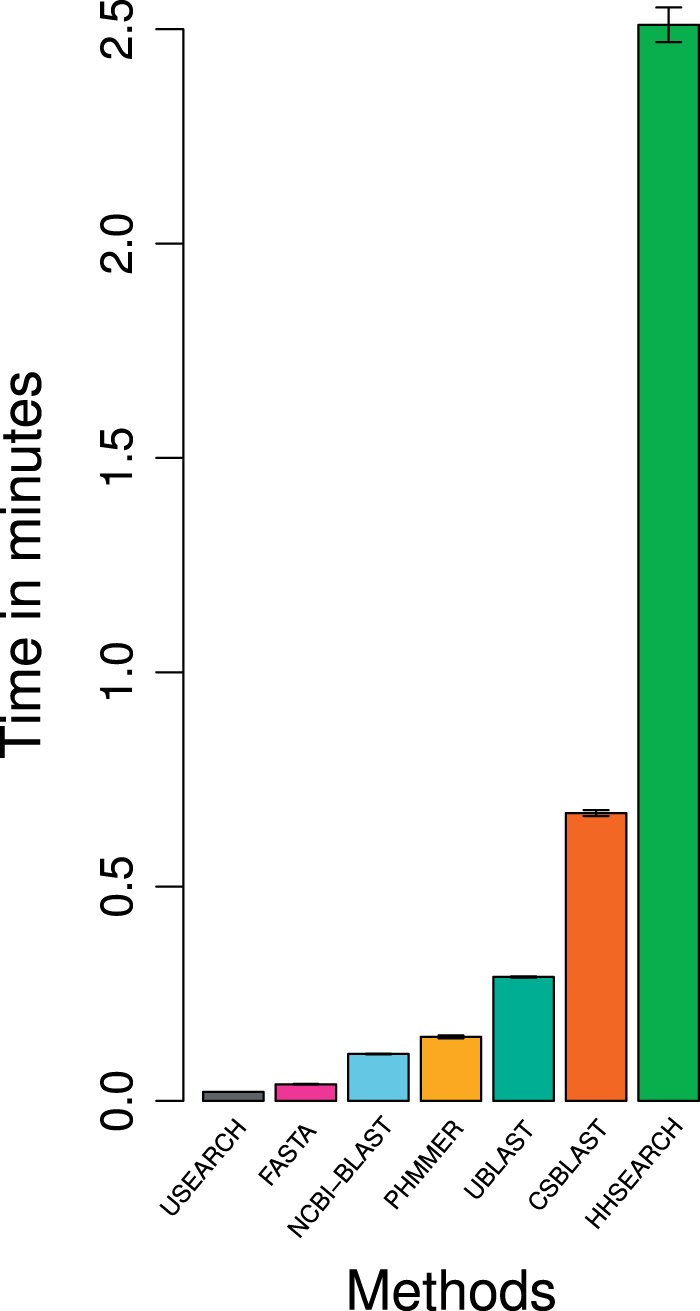
Run time for each homology inference method on a test set of 100 randomly sampled homologous protein pairs from the Pfam-based benchmark dataset, replicated ten times with different randomizations (mean and standard deviation shown as bars and error bars). All methods were run on an Intel Xeon E5540 @ 2.53 GHz with 24 GB RAM on a single core (Color version of this figure is available at *Bioinformatics* online.)

As a complement to benchmarking method accuracy, we also benchmarked run time by applying each tool to 100 randomly chosen protein pairs (repeated 10 times to achieve robust run time estimates), as shown in Figure 4. Profile-HMM methods were generally slower than heuristic string matching searches, with HHSEARCH taking the longest followed by CS-BLAST. PHMMER and NCBI-BLAST were intermediate, possibly due to speed being longtime development targets for both tools, and USEARCH and FASTA overall fastest. UBLAST is supposedly optimized for speed but ranked among the slower methods here. It should be noted that some methods may run faster on other hardware or in setups other than pairwise comparisons, e.g. by building a larger database and running multiple queries against it.

## 4 Discussion

Given the role of homology inference in genome-scale biology, validation and comparative benchmarking of the tools in use is important, even where it is difficult in both theory and practice to construct such benchmarks so that they will reflect the issues that may come into play in ‘live’ applications. We previously described an extensible strategy for such benchmarking and applied it to the then state-of-the-art of homology inference methods. In the present work, we have updated this approach and applied it to the ‘next generation’ of such methods. We have shown these benchmark results to be robust to the choice of underlying domain definitions, and we make the method available in script distribution for bioinformaticians seeking e.g. to optimize their particular analysis pipelines.

From our benchmark we observe that most profile methods have similar accuracy, with top performance from CSBLAST and the HMMER 3 protein search application PHMMER, whereas the speed-optimized FASTA and UBLAST/USEARCH are substantially less accurate. All profile-based methods outperform ‘classic’ single-sequence homology inference tools in terms of accuracy, but some of them do this with great sacrifice of speed.

Additionally, we show that the three most widely used protein domain definition schemes are similar with regards to which conclusions on protein full-length homology or non-homology they lead to, implying that the differences between them with regards to source data, curation or methods chiefly lead to differences in coverage and granularity, but not so much to differences in what evolutionary entities end up classified as domain families. Consequently, analysis results from one generally transfers well to the others.

It is important to note that development of tools do not take place in a vacuum separated from curation and compilation of protein domain databases. It is therefore conceivable that currently unknown classes of protein folds exist where method performance is different. However, it is likely that most existing folds already are known (Roche and Brüls, 2015).

As stated previously, this benchmark leaves out recent developments (Boratyn *et al.*, 2012) that rely on information not contained within the query and database sequences. Such methods may improve performance beyond plain sequence comparison or iterative query tools. Evaluating them will however be a challenge for future benchmark efforts since they depend on additional data beyond the family membership being tested.

## Supplementary Material

Supplementary Data
