# Supplementary material for: Benchmarking the next generation of homology inference tools
Source: Bioinformatics. 2016 Jun 1;32(17):2636–41. doi: 10.1093/bioinformatics/btw305 (PMC5013910; doi:10.1093/bioinformatics/btw305)
Supplement: Supplementary Data [file supp_32_17_2636__index.html]

Benchmarking the next generation of homology inference tools — Benchmarking the next generation of homology inference tools — Supplementary Data 

# Benchmarking the next generation of homology inference tools

## Supplementary Data

files

- Supplementary Data - zip file
