# Supplementary figures and images for: Benchmarking the next generation of homology inference tools
Source: Bioinformatics. 2016 Jun 1;32(17):2636–41. doi: 10.1093/bioinformatics/btw305 (PMC5013910; doi:10.1093/bioinformatics/btw305)

## A) PFAM + Clan

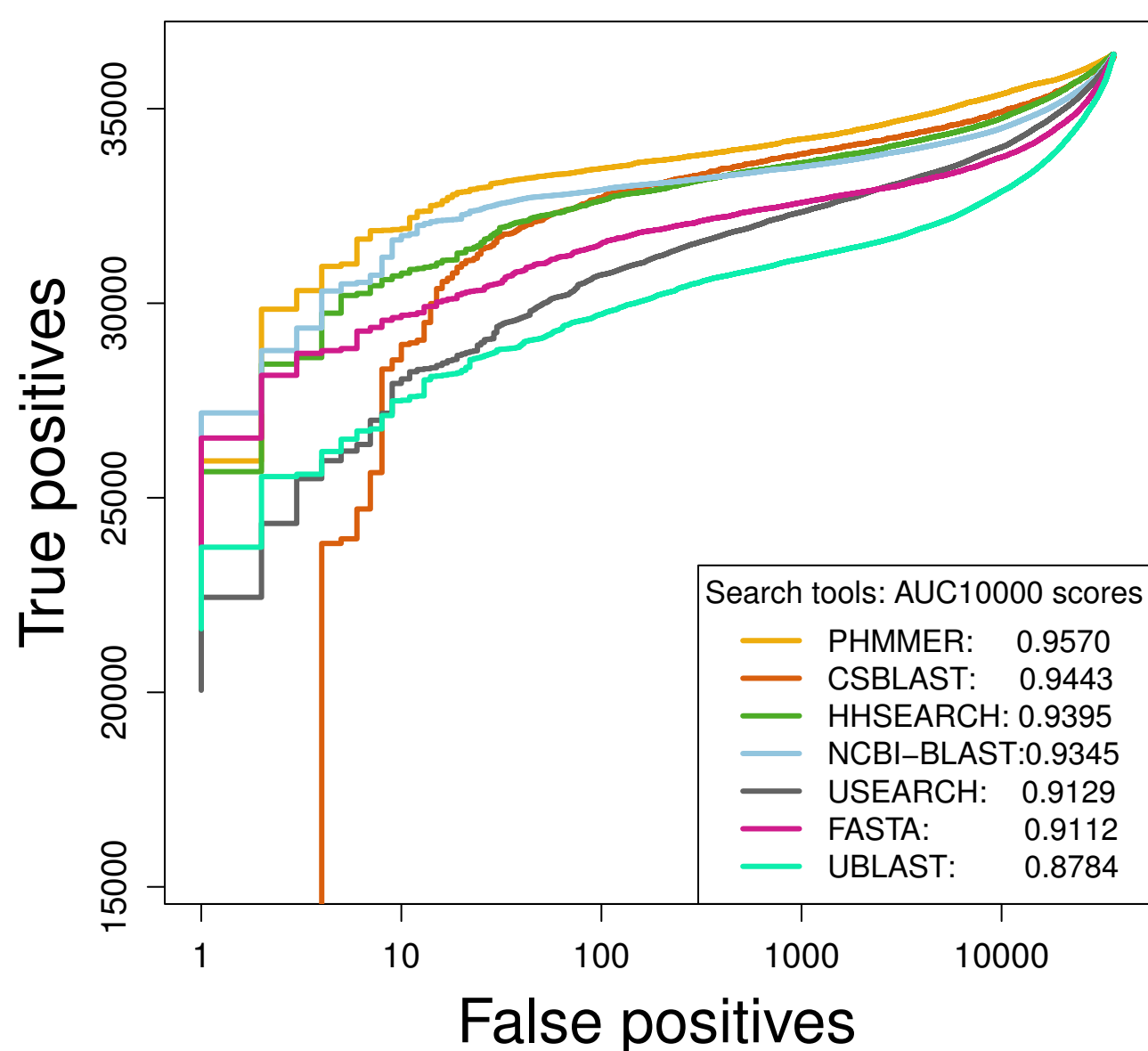

## B) SCOP/Superfamily

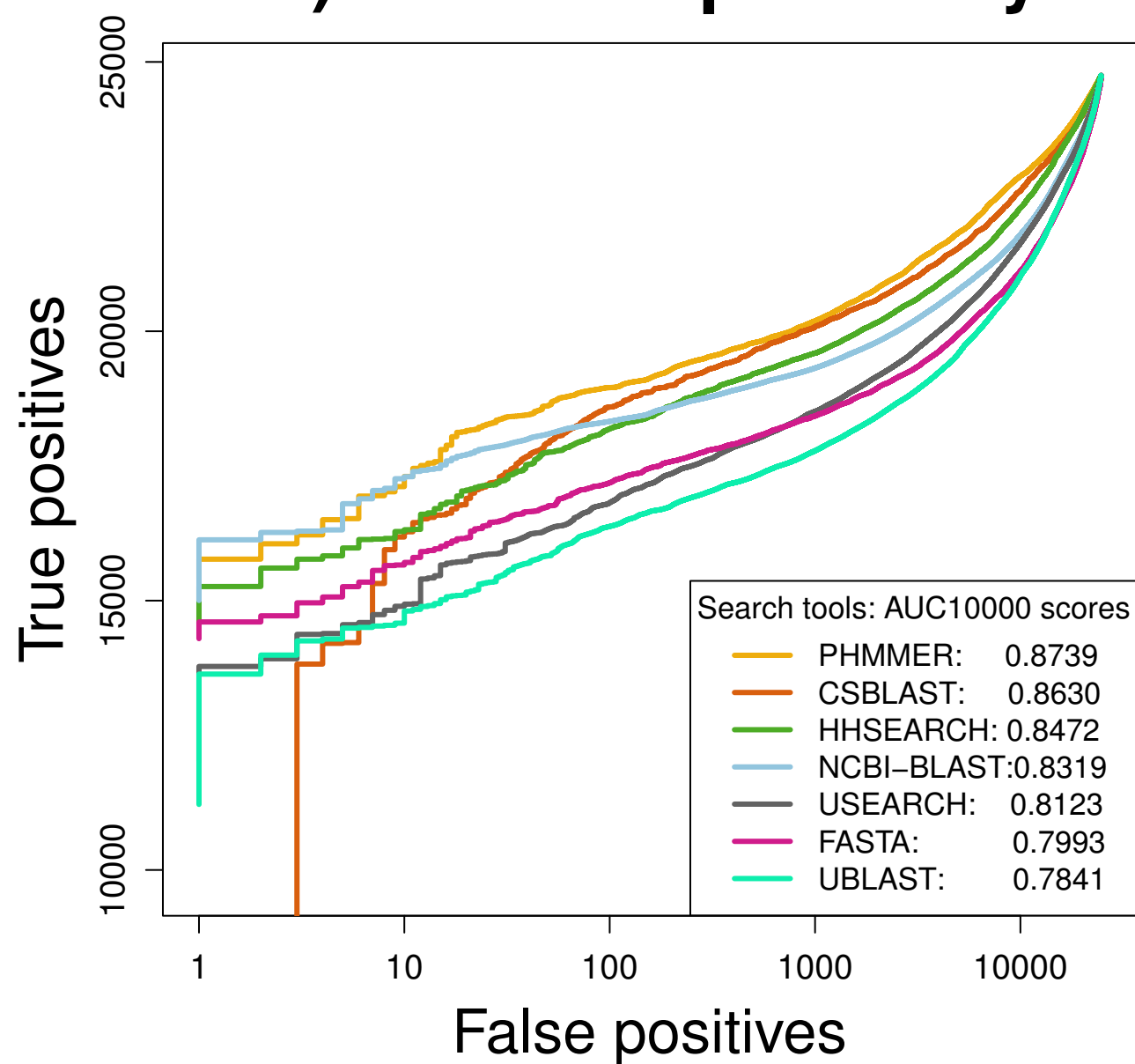

## C) CATH/Gene3D

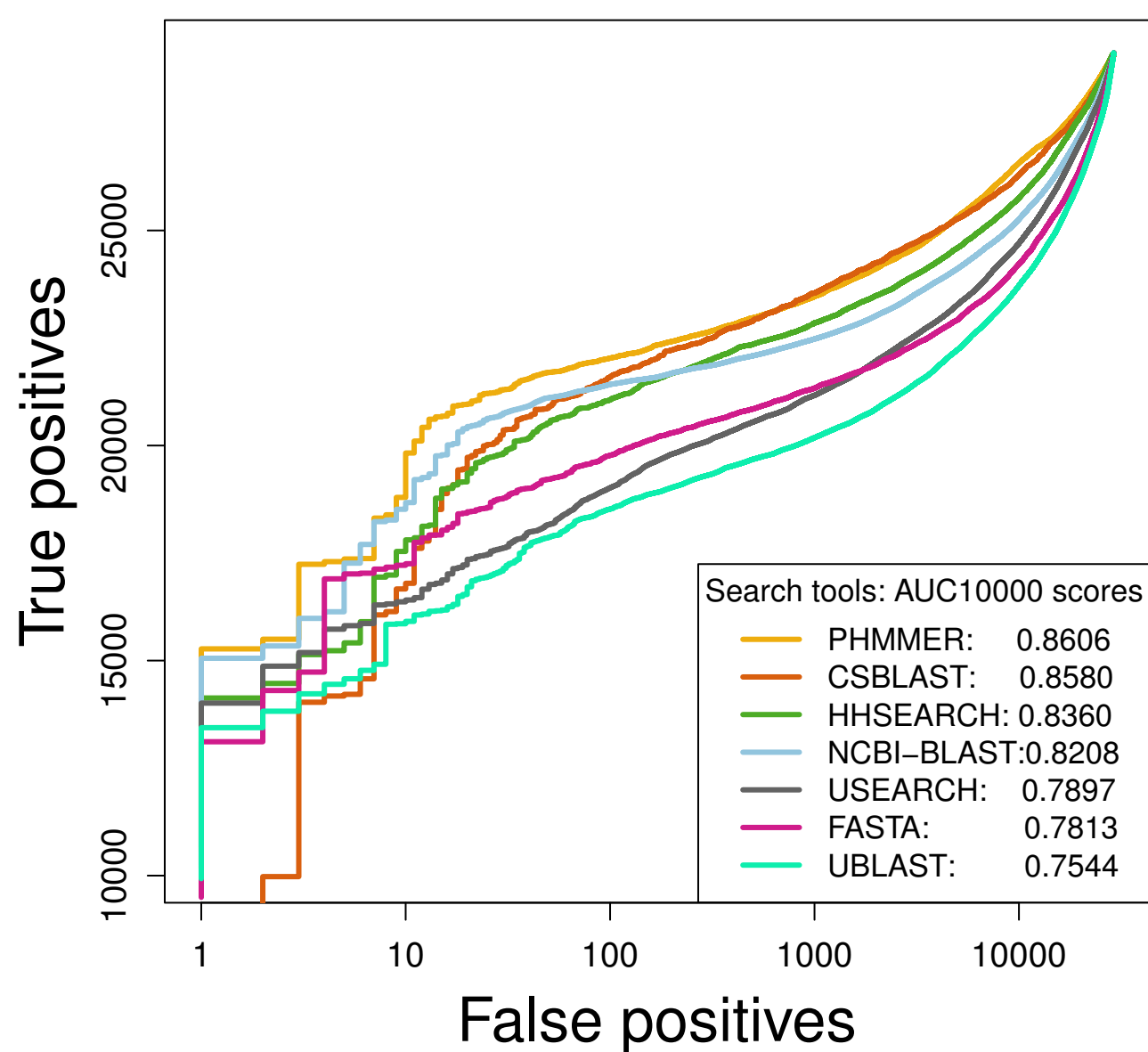

Supplement: Supplementary Data [file supp_btw305_suppl_data.zip › SupplementaryFigureS1.pdf]

Sensitivity

Specificity

Precision

Accuracy

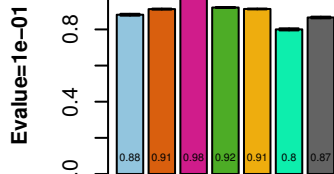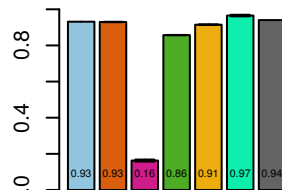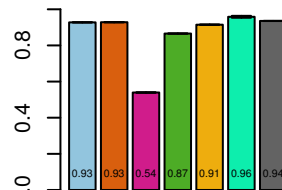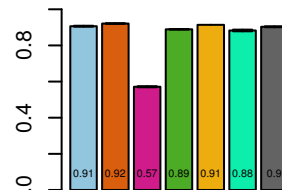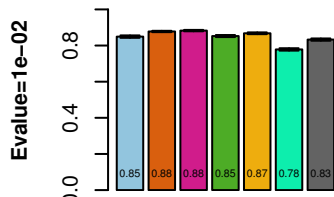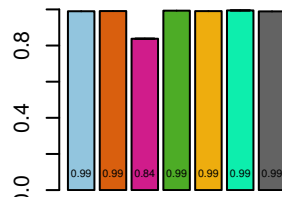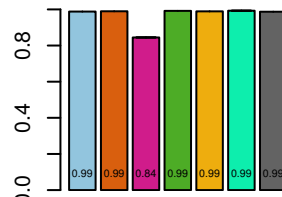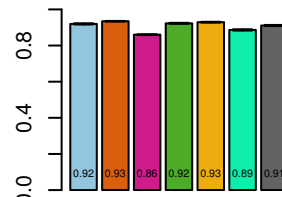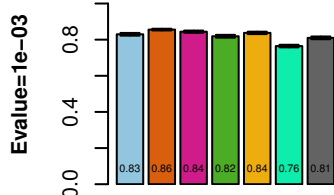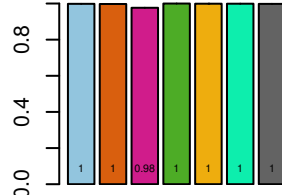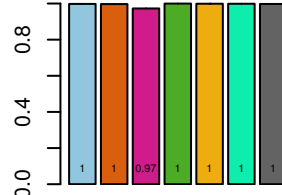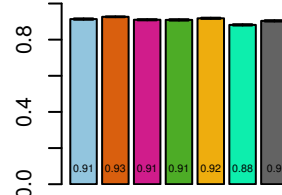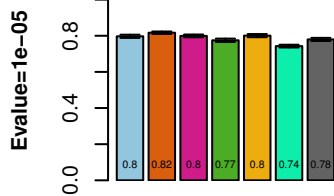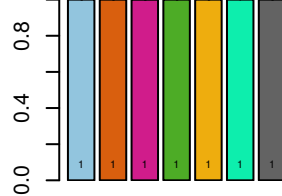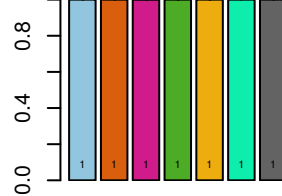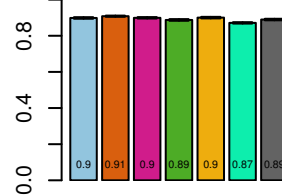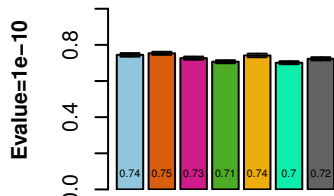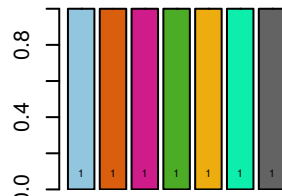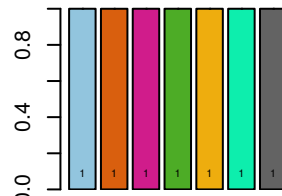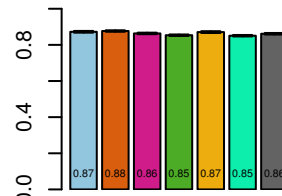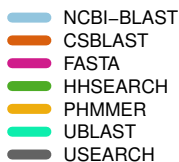

Supplement: Supplementary Data [file supp_btw305_suppl_data.zip › SupplementaryFigureS2.pdf]
