# Supplementary material for: Benchmarking the next generation of homology inference tools
Source: Bioinformatics. 2016 Jun 1;32(17):2636–41. doi: 10.1093/bioinformatics/btw305 (PMC5013910; doi:10.1093/bioinformatics/btw305)
Supplement: Supplementary Data [file supp_btw305_suppl_data.zip › SupplementaryTableS1.doc]

| S.No | Classification | Sub-classification | **Number of proteins** | **Species code** | **Species scientific names** |
| --- | --- | --- | --- | --- | --- |
| 1 | **A. Prokaryotes** | A.1 Archaea | 1026 | ARCFU A | Archaeoglobus fulgidus |
| 2 |  | A.2 Bacteria | 668 | ECOLX B | Escherichia coli (Gram-negative) |
| 3 |  |  | 318 | STAAU B | Staphylococcus aureus (Gram-positive) |
| 4 | **B. Eukaryotes** | B.1 Protists | 299 | CHLRE E | Chlamydomonas reinhardtii |
| 5 |  |  | 4131 | DICDI E | Dictyostelium discoideum |
| 6 |  | B.2 Fungi | 790 | EMENI E | Aspergillus nidulans (Emericella nidulans) |
| 7 |  |  | 6718 | YEAST E | Saccharomyces cerevisiae |
| 8 |  | B.3 Plants | 14095 | ARATH E | Arabidopsis thaliana |
| 9 |  |  | 753 | MAIZE E | Zea mays |
| 10 |  | B.4 Animals  B.4.1 Invertebrates | 3253 | DROME E | Drosophila melanogaster |
| 11 |  |  | 3596 | CAEEL E | Caenorhabditis elegans |
| 12 |  | B.4.2 Vertebrates | 20203 | HUMAN E | Homo sapiens |
| 13 |  |  | 2949 | DANRE E | Danio rerio |
| 14 |  |  | 3398 | XENLA E | Xenopus laevis |
| 15 |  |  | 2265 | CHICK E | Gallus gallus  Mus musculus |
| 16 |  |  | 16717 | MOUSE E | Mus musculus |

**Supplementary Table S1.** The subset of species used for the benchmark, selected for the purpose of spanning both short and long evolutionary distances as well as model organism utility, listed species have been selected from NCBI Taxonomy browser. Total protein counts were obtained from UniProt-SP.
