# Supplementary material for: Benchmarking the next generation of homology inference tools
Source: Bioinformatics. 2016 Jun 1;32(17):2636–41. doi: 10.1093/bioinformatics/btw305 (PMC5013910; doi:10.1093/bioinformatics/btw305)
Supplement: Supplementary Data [file supp_btw305_suppl_data.zip › SupplementaryTableS3.doc]

| **S. No** | **Version used for each method** | **Executable** | **Commands used for execution** |
| --- | --- | --- | --- |
| 1 | BLAST: ncbi-blast-2.2.31+ | makeblastdb | makeblastdb -in <seqfile1_db> -dbtype prot -out <databasefile> |
|  |  | blastp | blastp -query <seqfile2_query> -db <databasefile> -outfmt <5> -out <outputfile> -evalue <1000000000> -max_hsps 1 |
| 2 | CSBLAST: csblast-2.2.3_linux64 and blast-2.2.26 | formatdb | formatdb -p T -i <seqfile1_db> -n <databasefile> |
|  |  | csblast | csblast -i <seqfile2_query> -d <databasefile> -D <K4000.lib> -o <outputfile> --blast-path <blast-2.2.26/bin/> -e <1000000000> |
| 3 | FASTA: fasta-36.3.8b | fasta36 | fasta36 -q <seqfile2_query> <seqfile1_db> -m 8 -O <outputfile> -b <1000000000> |
| 4 | HHSEARCH: hhsuite-2.0.16-linux-x86_64 | hhmake | hhmake -i <seqfile1_db> -o <databasefile> |
|  |  | hhsearch | hhsearch -i <seqfile2_query> -d <databasefile> -o <outputfile> -E <1000000000> |
| 5 | PHMMER: hmmer-3.1b2-linux-intel-x86_64 | phmmer | phmmer –max -E <1000000000> –tblout <outputfile> <seqfile2_query> <seqfile1_db> |
| 6 | USEARCH: usearch8.1.1812_i86linux32 | -search_local | -search_local <seqfile2_query> -db <seqfile1_db> -evalue <1000000000> -blast6out <outputfile> |
|  |  | -ublast | -makeudb_ublast <seqfile1_query> -output <seqfile1_db.udb> |
|  |  | -ublast | -ublast <seqfile2_query> -db <seqfile1_db.udb> -evalue <1000000000> -blast6out <outputfile> |

**Supplementary Table S3.** The list shows all software tools tested with the specific parameters/settings used for each. All tools were run using default settings but with E-value report thresholds set to a high proxy (E-value = 1000000000) in order to report a score for as close to all comparisons of pairs as possible. The latest release versions of each tool was chosen.
