# Supplementary material for: Benchmarking the next generation of homology inference tools
Source: Bioinformatics. 2016 Jun 1;32(17):2636–41. doi: 10.1093/bioinformatics/btw305 (PMC5013910; doi:10.1093/bioinformatics/btw305)
Supplement: Supplementary file 7 [file supp_btw305_suppl_data.zip › SupplementaryTableS2.odt]

| S.No | Selected protein domain architectures | Excluding long unassigned regions | Including long unassigned regions |
| --- | --- | --- | --- |
|  |  | Pfam + Clan | SCOP | CATH | Pfam + Clan | SCOP | CATH |
| 1 | After species filter multi-domain proteins (redundant) | 3030 | 2489 | 2565 | 24326 | 14370 | 16653 |
| 2 | Removed motif-repeats and Clan-filter | 2998 | 2489 | 2565 | 23470 | 14370 | 16653 |
| 3 | Unique PMDAs well-defined in all three domain databases | 791 | 535 | 496 | 5266 | 2684 | 2476 |
| 4 | Unique PMDAs, selected two proteins per species per UPMDA | 455 | 330 | 339 | 3213 | 1578 | 1513 |
| 5 | Total proteins derived for benchmark | 1872 | 1560 | 1645 | 13424 | 7434 | 7731 |
| 6 | Total protein pairs derived for benchmark | 5245 | 5047 | 5656 | 36401 | 24751 | 29131 |

Supplementary Table S2.   Total number of protein pairs derived for the benchmark from the three different domain databases. To reduce the total number pairs we have selected two proteins per species per Unique Protein Multi Domain Architecture (UPMDA).
